# What factors are associated with patients walking fitness when starting cardiac rehabilitation?^☆^

**DOI:** 10.1016/j.ijcha.2018.11.002

**Published:** 2018-11-23

**Authors:** Martina Minotto, Alexander S. Harrison, Giovanni Grazzi, Jonathan Myers, Patrick Doherty

**Affiliations:** a University Degli Studi Di Ferrara, Italy; b University of York, UK; c Palo Alto Health Care System, United States of America

**Keywords:** Heart failure, Physical fitness, Cardiac rehabilitation, Incremental shuttle walk test

## Abstract

**Introduction:**

Patients with heart failure (HF) are now a priority group for cardiac rehabilitation (CR). A fundamental component of CR is increasing physical fitness through exercise training. Even though studies show fitness benefits, there is little evidence in routine populations of individual factors that may influence fitness. This study aims to evaluate the extent to which demographics and clinical measures predict physical fitness in patients with HF and develop reference values to guide practice.

**Methods:**

Data from the National Audit Cardiac Rehabilitation (NACR) was analysed. 2047 patients (73% male) with HF completed an incremental shuttle walk test (ISWT). Backward regression accounting for patient characteristics and new comorbidity groups were used to identify predictors of distance using ISWT. Reference values were produced from the percentiles of the ISWT distance.

**Results:**

Population age was 64.43 years (12.39 SD) with an average ISWT distance of 278.57 m (SD 158.57). Demographics, risk factors and comorbidities explained 26% of the variance in distance (adjusted R^2^ = 0.256, *p* value < 0.001). Diuretics (−33.01 m ±8.07 SD) and socioeconomic status (9.12 m ±2.91 SD) were significant predictors of baseline walking fitness. Furthermore, respiratory obstructions, musculoskeletal issues and metabolic diseases were associated with reduced walking distance of 29.8 m, 26.6 m and 18.4 m respectively.

**Conclusion:**

Use of diuretics, socioeconomic status and presence of comorbidities were significant predictors of walking performance in patients with HF who attended CR and were fit enough to carry out an ISWT. Reference values, to aid clinical practice, were developed that included age, gender and comorbidities status.

## 1 Introduction

Heart failure (HF) is a common chronic cardiovascular condition. According to the BHF CVD Statistics, over half a million people in the UK have been diagnosed with HF [1]. Patients with HF experience marked reductions in their exercise capacity or physical fitness which has detrimental effects on their activities of daily living, health-related quality of life and ultimately their hospital admission rate and mortality [2]. In addition to their heart condition, patients with HF often have the presence of other chronic comorbidities which may further reduce their physical fitness.

Cardiac rehabilitation (CR) is a safe and effective intervention in the management of cardiovascular diseases (CVD), which improves physical fitness, recovery and psychological well-being [3]. A critical part of the CR programme is exercise training [4]. Based on several decades of evidence, including a recent Cochrane review [5], the American College of Cardiology/American Heart Association, European Society of Cardiology and National Institute for Health and Care Excellence (NICE) [[6], [7], [8]], CR is now highly recommended for HF patients. In accordance with the recent 3rd edition of BACPR guidelines, this population is listed as a priority group who should receive exercise-based CR as secondary prevention [9].

Improvements in the referral rate of patients with HF to CR have occurred in recent years, increasing from 7% to 20% from general or cardiology wards. NICE guidance recommends increasing uptake to CR through improved recruitment of patients with HF to CR by offering tailored modes of exercise delivery led by a multidisciplinary team [8].

Although clinical trial data on HF exists indicating a relationship in terms of improving maximal exercise capacity through CR. The available studies tend to be based just on cardiopulmonary exercise testing (CPET) results among unique volunteer populations largely represented by relatively younger males, with fewer comorbidities compared to patients who participate in CR in routine practice [10,11].

Increasingly sub-maximal field tests are being used in clinical practice with the incremental shuttle walking test (ISWT) being the most common test in the routine UK CR [10]. It is reliable and strongly correlated with CPET results validated in cardiac and pulmonary populations undergoing rehabilitation programmes [[12], [13], [14], [15]]. Some studies have used the ISWT to investigate potential determinants of walking fitness in conventional cardiac populations [16,17] identifying several personal characteristics as significant predictors of fitness, including gender, age, body mass index, physical activity status, employment and marital status [15,17,19]. The impact of comorbidities, which often occur together with the diagnosis of HF, particularly in elderly patients, have not yet been investigated for their impact in determining walking fitness in terms of distance achieved using the ISWT.

The current study aims (1) to investigate and identify, among patients with HF, any relevant associations between walking fitness and significant patient characteristics, risk factors and comorbidities, conceptualized in a new classification based on similar clinical type, and (2) to produce HF specific reference values in terms of distance achieved using the ISWT.

## 2 Methods

### 2.1 Data collection

The analysis used anonymised data routinely collected by the NACR for patients undertaking CR in England [10]. The electronic data were acquired from 224 programmes which collect information on patients, including initiating event, demographic details, risk factors and comorbidities, treatments, medications and clinical outcomes pre- and post-CR. This study included patients who have a diagnosis of HF and were referred for CR between 1st January 2013 and 30th April 2018.

Maximum walking distance (in metres) performed during an ISWT, as a baseline measure of physical fitness recorded during a pre-CR assessment, represents the dependent variable. The ISWT is a recommended sub-maximal, incremental, externally paced test widely-used to evaluate exercise capacity [9]: it has 12 levels each one characterised by a determined speed that increases from 1.9 km/h to 8.5 km/h every minute. The patient is required to walk along a 10 m course following the external pace imposed until the end or the stop of the test [[12], [13], [14], [15]]. The ISWT outcome was analysed in terms of total metres walked which, as a continuous variable, naturally takes account of the incremental speed levels. This approach is consistent with robust linear regression models and also enabled us to create reference values which are more understandable to clinicians and patients.

### 2.2 Statistical analysis

Group comparisons were performed using unpaired *t*-tests. Pearson correlation coefficients were used to determine the association between key continuous variables and ISWT performance. Backward linear regression models were created to determine which covariates were associated with fitness expressed as distance walked during the ISWT at baseline. The analysis accounted for 9 individual covariates and 7 comorbidity-categories.

Variables included in the analysis were based on literature evidence or relevance in the preliminary analyses. Age (years), gender (male/female), employment status (employed or retired/unemployed), marital status (single/partnered) and ethnicity (White/Non-White – which includes Indian and Black Caribbean) have previously been shown to influence outcomes in different CR programmes and were included in the analysis [15,17,19]. The use of diuretics (no/yes) was included and taking any loop or thiazide medications was recorded as being on diuretics, since they have similar effects. Furthermore, the Multiple Deprivation Index (IMD) status (an England only based score) was included in the study, investigating relevant influence on outcomes. Risk factors, including physical inactivity status at baseline based on the 150 min/week recommendation from the UK chief medical officer (no/yes), BMI and comorbidities were entered in the study regression model as they are routinely reported in the NACR annual report [10]. All patient's characteristics were included in the regression study as independent variables. Statistical level for significance was set at *p* value < 0.05; variables were included in the final regression model if they respect these criteria. The 25th, 50th and 75th percentiles of distance walked were developed to provide reference values. Statistical analysis was conducted using IBM statistical package SPSS V.25 (SPSS, Chicago, Illinois, USA).

## 3 Results

The study population included patients with HF who had attended a CR programme from England and who had undertaken an ISWT baseline assessment to evaluate physical fitness. The sample consisted of 2047 patients (73% male) with a mean age of 64.43 years (12.39 SD, range 19 to 98 years).

Table 1 shows the average ISWT distance in metres for each of the included variables. The overall mean distance was 278.57 m (158.57 SD). Pearson correlation coefficients indicate a significant negative relationship between age and ISWT distance (−0.379, *p* value < 0.001). In terms of gender, males covered a greater distance during the ISWT by an average of 63 m (*p* value < 0.001). Significant associations were observed between ISWT and physical activity status, BMI and use of diuretics (all *p* value < 0.001). Patients who achieved the weekly physical activity goal of 150 min/week (active subjects) achieved 66 m greater distance in comparison to those who did not achieve the 150 min/week threshold at moderate intensity. Patients with BMI >30 (overweight or obese) walked a shorter distance during the ISWT assessment by an average of 33 m less rather than those with BMI < 30. Patients who were taking diuretics achieve 55 m shorter distance versus those not taking diuretics. Furthermore, IMD status was significantly associated with ISWT (*p* value = 0.40) indicating that there was a gradient across the quintiles, with a greater ISWT performance from the lowest quintile compared to the 2nd quintile, by an average of 19 m. No other variables were associated with significant differences in ISWT distance.

**Table 1:** Incremental shuttle walk test (ISWT) distance for all included variables.

Patient characteristics	ISWT distance in metres [m]					
	Mean	SD	Count	%	Pearson correlation (PC)	*p* value
Age [years]	64.43	12.394	2047		−0.379	<0.001


Patient characteristics		ISWT distance in metres [m]					
Baseline demographics by distance [m]		Mean	SD	Count	%	Mean difference (MD)	*p* value
Gender	Male	294.91	163.65	1482	72.9	62.877	<0.001
	Female	232.03	133.03	552	27.1		
Ethnicity	White	276.85	156.71	1495	73.0	−6.376	0.42
	Non-White	283.22	163.55	552	27.0		
Physical activity status	No	257.82	153.35	1246	69.4	−65.688	<0.001
	Yes	323.51	153.01	550	30.6		
Body mass index (BMI)	BMI < 30	289.96	162.68	1182	61.8	33.327	<0.001
	BMI > 30	256.64	145.75	731	38.2		
Diuretics	No	305.84	168.99	1033	50.5	55.053	<0.001
	Yes	250.78	141.99	1014	49.5		
Employment status	Employed	272.89	153.61	1335	77.8	−12.841	0.151
	Unemployed	285.73	154.08	381	22.2		
Marital status	Single	270.17	170.21	687	39.3	−9.161	0.233
	Partner	279.33	147.81	1062	60.7		
Total population ISWT score		278.57	158.57	2047			


Patient characteristics		ISWT distance in metres [m]					
		Mean	SD	Count	%	F	*p* value
IMD	Lowest quintile	260.47	169.15	368	22.3	1.004	0.404
	2nd quintile	280.27	161.13	361	21.9		
	3rd quintile	278.46	154.95	312	18.9		
	4th quintile	276.63	158.27	314	19		
	5th quintile	281.01	154.61	295	17.9		
	Total	274.95	160.14	1650	100		


Patient characteristics		ISWT distance in metres [m]					
		Mean	SD	Count	%	MD	*p* value
IMD	Lowest quintile	260.47	169.15	368	22.3	18.641	0.049
	≥2nd quintile	279.11	157.28	1282	77.7		

Seven alternative categories for grouping comorbidities were created and included in this study based on the 18 comorbidities captured by NACR data. Table 2 shows group comparisons between each of the comorbidity categories and ISWT walking distance [m]. Patients included in the category Ischemia+, Musculoskeletal Metabolic Hypertension and COPD + Asthma perform significantly lower on the ISWT. The mean difference in ISWT performance ranged from 36 and 65 m (all *p* value < 0.001) between those who experienced an ischemic event and those who had musculoskeletal comorbidities, respectively.

**Table 2:** Group comparison between comorbidity groups and ISWT distance [m].

Comorbidity group		ISWT distance in metres [m]					
		Mean	SD	Count	%	Mean difference (MD)	*p* value
Ischemia+	No	284.87	161.58	1686	82.4	35.769	<0.001
	Yes	249.11	140.14	361	17.6		
Psychosocial	No	280.15	159.27	1782	87.1	12.233	0.241
	Yes	267.92	153.65	265	12.9		
Musculoskeletal	No	295.16	160.42	1528	74.6	65.465	<0.001
	Yes	229.70	142.27	519	25.4		
Metabolic	No	292.92	164.75	1333	65.1	41.138	<0.001
	Yes	251.78	142.62	714	34.9		
COPD + Asthma	No	285.44	159.51	1750	85.5	47.400	<0.001
	Yes	238.04	146.74	297	14.5		
Hypertension	No	292.67	165.53	1299	63.5	38.584	<0.001
	Yes	254.08	142.51	748	36.5		
Erectile dysfunction	No	279.78	159.59	1919	93.7	19.455	0.179
	Yes	260.33	141.58	128	6.3		

Results from the linear regression between ISWT performance and patient characteristics, risk factors and comorbidities are shown in Table 3. The regression model indicates that age is negatively correlated with ISWT walking distance (B = −5.12, ±0.38 SE, *p* < 0.001). For each year of increased age above the mean, there was a 5.12 m decrease in distance covered during the ISWT. Gender was also a strong determinant of walking fitness; within the model, females achieved an average of 49.41 m (±9.48 m SE) less than their male counterparts (*p* < 0.001). Other statistically significant associations with ISWT distance included physical activity, BMI, employment, marital status and use of diuretics. HF patients who did not achieve the minimum weekly recommended amount of physical activity, those with higher BMI, unemployed, not in a relationship and taking diuretics were all associated with a lower ISWT score, ranging between 23 and 52 m (*p* 0.007 to <0.001). In addition, IMD status had a significant and positive association with ISWT performance (*p* value = 0.002). There was an increase of 9.12 m (±2.91 SE) in ISWT performance for each increment of socioeconomic status expressed by IMD quintiles. Between all the new studied comorbidity groups, only *Musculoskeletal Metabolic* and *COPD* *+* *Asthma* were significantly associated with ISWT distance (*p* 0.032–0.004). The combined comorbidity of *COPD* *+* *Asthma* were the strongest determinant of walking fitness (B = −29.79, ±11.19 SE).

**Table 3:** Linear regression findings for the ISWT by patient characteristics.

Patient characteristics	B	Std. error	*t*	Sig.	95% confidence interval	
					Lower bound	Upper bound
Age	−5.115	0.376	−13.587	<0.001	−5.854	−4.376
Gender (female)	−49.409	9.476	−5.214	<0.001	−68.003	−30.815
Physical activity status (150 min/week)	52.233	8.677	6.020	<0.001	35.208	69.258
BMI (>30)	−34.212	8.561	−3.996	<0.001	−51.009	−17.414
Employment status (unemployed)	−47.231	10.862	−4.348	<0.001	−68.545	−25.917
Marital status (partnered)	22.616	8.392	2.695	0.007	6.149	39.084
Diuretics (yes)	−33.059	8.071	−4.096	<0.001	−48.895	−17.223
IMD status	9.120	2.907	3.138	0.002	3.417	14.823
Musculoskeletal group (yes)	−26.640	9.335	−2.854	0.004	−44.957	−8.323
Metabolic group (yes)	−18.357	8.561	−2.144	0.032	−35.156	−1.559
COPD + Asthma (yes)	−29.791	11.185	−2.663	0.008	−51.739	−7.844
Constant	623.389	27.27	22.86	<0.001	569.88	676.897

R = 0.513; R^2^ = 0.264; Adj R^2^ = 0.256.

Ethnicity and other comorbidities were not significantly associated with ISWT distance, thus they were automatically removed from the backward regression analysis. The model residuals met the assumptions of uniform variance, linearity, with an adjusted R squared value of 0.256 (*R* = 0.513).

Table 4 shows reference values for patients able to carry out the ISWT, stratified by age (≤65 years old–65+ years old), gender and comorbidity groups displayed in 5th, 25th, 75th and 95th percentiles.

**Table 4:** ISWT reference values for HF patients according to age, gender and presence of any significant comorbidity groups.

Patient characteristics			Mean	SD	Percentile 05	Percentile 25	Percentile 75	Percentile 95	Count
Young HF ≤65	Male	COPD + Asthma	302.17	166.45	70	180	375	670	88
		Metabolic	313.49	160.19	80	180	420	630	230
		Musculoskeletal	301.15	173.49	30	180	420	630	128
		None-of-the-above comorbidities	377.65	180.46	80	250	520	640	356
	Female	COPD + Asthma	224.44	141.91	60	90	320	520	36
		Metabolic	228.14	127.68	60	130	300	520	59
		Musculoskeletal	216.76	113.94	60	120	305	420	68
		None-of-the-above comorbidities	310.39	150.06	70	210	420	550	140
Old HF 65+		COPD + Asthma	226.30	126.95	50	120	320	490	125
		Metabolic	235.91	126.97	60	150	330	420	325
		Musculoskeletal	220.20	133.08	50	120	280	450	210
		None-of-the-above comorbidities	263.90	144.54	70	170	350	520	291
	Female	COPD + Asthma	149.35	98.19	30	70	180	350	46
		Metabolic	167.54	88.42	40	100	220	330	95
		Musculoskeletal	174.21	97.26	40	90	240	340	113
		None-of-the-above comorbidities	226.94	118.83	50	140	310	420	121

Based on the strength of findings from the regression, Table 4 sets out new reference values.

## 4 Discussion

This study aimed to define the extent by which demographic and clinical variables predicted walking fitness in patients with HF, additionally, to define reference values to guide practice.

The mean score that patients with HF achieved during the ISWT is similar to the 25th percentile of conventional CVD patients with same mean age attending CR [15]. It is likely that the reason why these patients are capable of achieving comparable ISWT results despite their HF condition and the presence of additional comorbidities, is that the sample is mostly represented by patients with HF classes NYHA I and II (63%).

Based on the literature, age and gender have an unfavourable impact on walking fitness [15,17,19]. After accounting for the presence of new-grouped-comorbidities, each one year above the mean age (64.43 years ± 12.39 SD) and being female were significantly associated with a reduced walking performance of 5.12 m and 49.41 m, respectively.

Another important determinant in walking fitness is the physical activity status: the regression shows that achieving the weekly goal of 150 min of physical activity at moderate intensity is significantly positive associated with an increased walking distance at the ISWT by an average of 52.23 m (8.68 SD). The analysis also concludes that other variables, including BMI (>30), employment status (unemployed) and marital status (single) are inversely correlated with walking fitness. However, the impact of diuretics and IMD status are novel findings in this population. According to the regression model, taking diuretics is associated with poorer physical fitness, with a walking distance reduction by an average of 33.06 m (8.07 SD). This reduced fitness level may not be directly related to diuretics as it could be influenced by other factors such as being overweight or obese and HF severity. For instance, the proportion of overweight or obese patients was greatest in NYHA class IV as was the proportion of patients taking diuretics. We also assessed the extent to which socioeconomic status might influence physical fitness among patients with HF. The IMD status was a significant predictor of ISWT performance; a higher socioeconomic status was associated with a higher ISWT distance. We observed a 9.12 m (2.91 SD) higher walking distance per quintile IMD. The largest difference in walking distance was observed between the least two quintiles versus the 5th. This suggests that living in an area with a high score of deprivation is associated with a poor walking distance.

The concept of grouping comorbidities based on similar clinical categories appears to be a novel method of understanding the complexity of HF associated physical fitness. We tended to group under the same category *Musculoskeletal* comorbidities such as arthritis, osteoporosis, rheumatism and chronic back pain since they share a common inflammatory or chronic process that involves muscles and joints; *Metabolic* included any type of comorbidity that resulted in metabolic impairment, including diabetes and hypercholesterolemia/dyslipidemia; and the *COPD + Asthma* category any kind of pulmonary obstruction. The presence of any one of these comorbidity categories was significant in predicting walking fitness in patients with HF. The most debilitating comorbidity was *COPD + Asthma*, which was associated with a 29.79 m (±11.19 SD) reduction in walking distance. Presence of pulmonary obstruction (irreversible or reversible) results in the symptom of breathlessness that generally occurs with exertion or at rest when the condition is severe (COPD) [18]. There were also significant inverse associations with the presence of any *Musculoskeletal* condition, which reduced walking performance in the order of 26.64 m (±9.34 SD), and *Metabolic comorbidities* which was associated with an 18.36 m (±8.56 SD) reduction in walking distance. Both of these comorbidities, which often are exclusion criteria for clinical trials involving exercise, clearly add to the burden that impairs walking ability in patients with HF [5].

Since the presence of this comorbidity, together with demographic characteristics, significantly influence fitness of patients with HF, we developed tables with reference values categorized by age and gender that help to better understand the walking capabilities among HF patients participating in CR, taking into account the impact of different comorbidities. This represents a new approach which accounts for the presence of comorbidities accompanying HF which should enable clinicians to better understand the exercise capabilities of a given patient and to assist with the development of an appropriate exercise prescription during CR.

Our findings have generated new knowledge to guide clinical decisions regarding the suitability of patients with HF and their ability to participate in CR. The creation of reference values is suggested to guide the CR team in developing individualized exercise programmes that are appropriate for a given patient's condition, age, gender and presence of any comorbidities, and based on their walking capacity at the beginning of CR.

## 5 Limitations

Although this study investigated a large sample of patients, the ISWT results are not fully representative of the broader severity of HF typical of CR programmes. Due to the predominance of NYHA class II (47.7%) and the small proportion of patients in NYHA class III (25.8%) and IV (11.3%), the analysis could be biased despite the consideration of comorbidity status. Furthermore, although the use of diuretics was a significant predictor of walking fitness, we were unable to account for the dosage of these medications because the NACR does not capture this information. Diuretics was associated with NYHA class, however, due to insufficient sample size in the NYHA variable this interaction could not be included in the regression and will assessed in future work.

## 6 Conclusion

The study has concluded that use of diuretics, IMD status and the presence of *COPD* *+* *Asthma*, *Musculoskeletal* and *Metabolic comorbidities* are significant characteristics strongly associated to walking fitness in patients with HF. The creation of reference values for patients with HF makes this study unique in that it clarifies the extent to which individual factors and presence of comorbidities determine walking capability in terms of distance walked on a baseline ISWT assessment. The development of these reference tables permits a better understanding of the functional capabilities of a patient with HF at the initiation of CR programme, and to optimize an appropriate and individualized exercise-based intervention for a given patient.

## Conflict of interest

The authors report no relationships that could be construed as a conflict of interest.
